# *Lactobacillus brevis* alleviates the progress of hepatocellular carcinoma and type 2 diabetes in mice model *via* interplay of gut microflora, bile acid and NOTCH 1 signaling

**DOI:** 10.3389/fimmu.2023.1179014

**Published:** 2023-05-10

**Authors:** Shujia Chen, Ping Han, Qian Zhang, Peiyan Liu, Jie Liu, Lili Zhao, Lianyi Guo, Jia Li

**Affiliations:** ^1^ Clinical School of the Second People’s Hospital, Tianjin Medical University, Tianjin, China; ^2^ Department of Hepatology, Tianjin Second People’s Hospital, Tianjin, China; ^3^ Department of Gastroenterology, First Affiliated Hospital of Jinzhou Medical University, Jinzhou, China

**Keywords:** type 2 diabetes, hepatocellular carcinoma, Lactobacillus brevis, gut microflora, bile acids, NOTCH 1 signaling, MMP9

## Abstract

Type 2 diabetes (T2DM) clinically exhibits a higher incidence of hepatocellular carcinoma (HCC), contributing to a lousy prognosis in patients harboring both diseases. Microflora-based therapy draws attention with low side effects. Accumulating evidence shows that *Lactobacillus brevis* can improve blood glucose and body weight of the T2DM mice model and reduce several cancer incidences. However, the therapeutic effect of *Lactobacillus brevis* in affecting the prognosis of T2DM+HCC remains unknown. In this study, we aim to explore this question *via* an established T2DM+HCC mice model. We observed a significant alleviation after the probiotic intervention. *Lactobacillus brevis* improves blood glucose and insulin resistance and ameliorates Mechanically. Combined with a multi-omics approach including 16SrDNA, GC-MS, and RNA-seq, we identified distinct intestinal microflora composition and metabolites after *Lactobacillus brevis* intervention. Furthermore, we found that *Lactobacillus brevis* delayed disease progression by regulating MMP9 and NOTCH 1 signaling pathways, potentially through gut microflora and BA interaction. This study indicates that *Lactobacillus brevis* may improve the prognosis of T2DM + HCC, providing novel therapeutic opportunities *via* targeting intestinal flora for patients with T2DM+HCC.

## Introduction

Type 2 diabetes (T2DM) is a common, chronic, multifaceted, and potentially fatal disease. Moreover, Patients with T2DM harbor increased morbidity and mortality of many malignant tumors (1). Insulin resistance, hyperinsulinemia, and insulin-like growth factor-1 (IGF-1) are critical factors in the carcinogenic mechanism (2). Hepatocellular carcinoma (HCC) is a widespread malignant tumor considered the leading cause of cancer-related deaths worldwide (3). Nowadays, it is known that T2DM is an independent risk factor for HCC (4). A retrospective review of HCC patients was conducted by Connolly et al. to assess the effect of T2DM on the clinical behavior of HCC. Distant metastasis was found in 33% of patients with T2DM compared with only 9.7% of those without T2DM (OR: 4.5, *P* < 0.0001). This difference remained significant also when adjusting for other clinical variables (5). Unfortunately, studies using mice models to explore the therapeutic options for T2DM+HCC are minimal. Previously, amounting evidence indicated that gut microbiota (GM) plays a vital role in affecting HCC carcinogenesis and drug response in both mice models and patients (6, 7). Similarly, this phenomenon is also observed in T2DM. For instance, *Lactobacillus brevis* could improve the primary conditions of T2DM mice, such as blood glucose, and reduce the incidence rate of adverse events (8). However, the role of Lactobacillus brevis in T2DM patients with HCC is unknown.

Among various human body metabolites processed by GM, bile acids (BAs), synthesized mainly in the liver, have attracted more and more attention because of their known tumor-promoting properties (9). Besides, T2DM patients with high BAs tend to have more severe metabolic disorders (10). Given the known interaction among GM-BA, it would be interesting to investigate the role of GM-BA interactions in affecting T2DM+HCC disease progress.

In this study, we aim to explore the therapeutic effect of *Lactobacillus brevis* on mice harboring T2DM+HCC through the BAs-GM axis to affect disease progression. We systematically analyze the GM composition and metabolic changes in T2DM+HCC mice models and identify the significant difference in GM diversity and Bas levels induced by *Lactobacillus brevis* treatment. This will provide new strategies to improve the prognosis of patients with T2DM+HCC through GM and metabolic intervention.

## Materials and methods

### Experimental design

Male C57BL/6 mice were purchased from Weishanglide Biotechnology Co., Ltd, China. The animal experiment ethics committee approved the experimental animal study protocol (VS212600898). Experimental animals were used following the Guide for the Care and Use of Laboratory Animals established by the National Institutes of Health (11). The mice were housed under a 12 h light/dark cycle in a constant temperature and humidity-controlled environment (20± 2°C and 60%, respectively), with free access to food and water. All mice were acclimatized to the housing environment for 7 days before any experiment.

To establish a mice model bearing T2DM+HCC, we followed the procedures Kang (12) et al. reported. Detailly, for diabetes induction, male 18-20 g C57BL/6 mice aged 6 weeks were fed a 60% high-fat diet (Beijing HFK Biotechnology) for 3 weeks. On the 21st day, 50 mg/kg streptozotocin (STZ) was injected 5 consecutive days per week for 2 weeks. To validate the model’s success, the mice were fasting for over 6 hours before blood glucose measurement. The mice were considered to have the characteristics of T2DM with glucose values greater than 11.1 mmol/L. It is common in the T2DM mice model to control food intake/feeding patterns to ensure consistency and reproducibility of results. We achieved this using a standardized diet, careful measurement and providing food at specific times (8, 12, 20 h). To further establish the HCC model, the T2DM mice were intraperitoneally injected with diethylnitrosamine (DEN, 50 mg/kg, dissolved in saline) once on the 33rd day. On the 47th day, mice were intraperitoneally injected again with DEN (10 mg/kg). On the 35th and 54th days, CCl_4_ and olive oil (prepared in a volume of 2:3) were gavaged at 5 ml/kg. Meanwhile, mice drank water containing 9% ethanol on the 54th day. The growth, appetite, weight, behavior, and mental status were observed during the experiment. In the total period of around 21 weeks, H&E staining was used to confirm the liver change of HCC pathology.

The mice were divided into 6 groups, with 15 in each group. For 10 mice in health control (HC), mice were given *ad libitum* access to food and water. For the 50 mice in the disease group, 10 mice in the vehicle group (Veh) were orally administered PBS once a day; 10 mice in acarbose (ACA, CatNo. HY-B0089, Solarbio) group were orally administered with 50 mg/kg once a day; For the probiotic group, 30 mice were given live *Lactobacillus brevis* (CatNo. 20297, Solarbio) by oral gavage from low dose (LD, 10^8^ cfu/mL), through median dose (MD, 10^9^ cfu/mL) to high dose (HD, 10^10^ cfu/mL).

### Glucose tolerance test (GTT)

Mice were fasted for 16 h with water available, and weight and blood glucose were measured. After 30 minutes, glucose (CatNo. A8760, Solarbio) was injected intraperitoneally at 2 g/kg. Blood samples were taken 15, 30, 60, 90, and 120 minutes after injection to measure blood glucose levels. The blood was collected from the tail tip, cut off 1-2 mm every time. The first drop was discarded and the second was used to measure blood parameters.

### Insulin tolerance test (ITT)

Mice were fasted for 6 h with water available, and weight and blood glucose were measured. After 30 minutes, insulin (CatNo. RAB0904, Sigma) was injected intraperitoneally at 1 U/kg. Blood samples were taken 15, 30, 60, 90, and 120 minutes after injection to measure blood glucose levels.

### RNA extraction and quantitative real-time PCR

Total RNA was extracted from liver tissue by Trizol reagent (CatNo. R1100, Solarbio) and was reversely transcribed to cDNA according to the manufacturer’s protocol (HiScript II Q RT SuperMix, CatNo. R223-01, Solarbio). Primers were designed and purchased from Solarbio and quantitative PCR was further performed following the manufacturer’s protocol (Novozan AceQ qPCR SYBR GreenMaster Mix, CatNo. Q111-02, Solarbio) using LightCycler96 real-time fluorescence quantitative PCR instrument (CatNo. 05815916001, Roche)(**Supplementary Table 1**).

### Western blotting

The liver tissue was lysed in RIPA buffer (CatNo: R0010, Solarbio), and the total protein concentration was determined using a BCA kit (CatNo. 23225, Thermo Scientific). 30 μg protein per lane were separated from a 6-15% sodium dodecyl sulfate-polyacrylamide gel (CatNo: 28312, Thermo Scientific) and further transferred to a polyvinylidene fluoride (PVDF) membrane (CatNo: 24585 Thermo Scientific). The membrane was blocked with 5% BSA for one hour. The membrane was then incubated overnight at 4°C with primary antibodies against MMP9 (1:500, CatNo: ab76003, Abcam), NOTCH 1 1 (1:1000, CatNo: ab52627, Abcam), Hes1 (1:1500, CatNo: ab108937, Abcam), Math1 (1:1000, CatNo, ab249467, Abcam), and GAPDH (1:10000, CatNo: 14C10, Cell Signaling Technology). After three washes with TBS buffer containing 0.1% Tween-20 (TBST), the membrane was incubated with secondary antibodies conjugated horseradish peroxidase (CatNo: ab2116, Abcam) for one hour. After three times washing with TBST, a HyGLO-HRP kit (CatNo: 32106, Thermo Scientific) was used to visualize the bands.

### Enzyme-linked immunofluorescence assay (ELISA)

Mice underwent orbital blood collection. After centrifuging at 3000 rpm for 10 min, the supernatant was collected, and the TBA (Beyotime, CatNo: SC3319-5mg), LPS (Beyotime, CatNo: S1732-5mg), and TMAO (Shanghai Hengyuan Biology, CatNo: HB150 Mu) levels were detected using an ELISA kit according to the manufacturer’s instructions.

### Detection of intestinal micro-organisms and bioinformatics analysis

The V3 and V4 sequences of 16SrDNA from intestinal bacteria in mice feces were determined by high throughput (MEJI Biotech sequencing, China). The original data obtained from sequencing are first spliced to make the results more accurate and reliable. The sequence quality is filtered through quality control to get clean data OTUs (operational, economic units) clustering and species classification was conducted with 97% similarity as the threshold. With the help of the I-Sanger cloud platform, according to the OTUs clustering results, the representative sequences of each OTU are annotated with species. The corresponding species information and abundance distribution, sample comparison, model prediction, 16S function, and pathway prediction analysis are obtained. The original sequence data was stored at National Center for Biotechnology Information (NCBI, https://www.ncbi.nlm.nih.gov/).

### Hematoxylin-eosin staining(H&E)analysis

The liver and pancreatic tissues were fixed with formalin and embedded in paraffin, followed by cutting into 4 µm. The slides were dewaxed and gradient treated with xylene and ethanol for hematoxylin and eosin (H&E) staining (CatNo. G1120, Solarbio) and immunoperoxidase staining.

### Immunofluorescence

The liver tissues of 6 groups of mice were fixed and sliced, with a thickness of about 4 μm. After antigen recovery, the slice was incubated with the primary antibody Ki-67 (1:50, CatNo: K009725P, Solarbio) diluted in PBS containing 3% albumin bovine V (CatNo. R4903, Solarbio) at 4 °C and then incubated with the secondary antibody diluted in PBS at room temperature for 60 min. After nuclear staining with DAPI (CatNo: C1002, Beyotime), the slides were mounted with a fade-resistant mounting medium. LSM 510 confocal microscopes were used to analyze immunofluorescence staining sections.

### Clinical data and sample collection

The patients enrolled in this study were from two medical centers, the Second People’s Hospital of Tianjin and the First Affiliated Hospital of Jinzhou Medical University, during 2016-2022. The Ethics Committees from both centers approved this study. Each patient was informed with written consent. T2DM was diagnosed based on the World Health Organization (WHO) recommendation in 1999 (13). The diagnostic criteria for HCC were from the guideline of the European Association for the Study of the Liver (14). Patients were excluded if they had (i)Type 1 diabetes and other types of diabetes, (ii) acute complications of diabetes; (iii) disorders of thyroid, rheumatism and other chronic diseases, (iv) a drug history of glucocorticoids or antibiotics, (v) mental illness, (vi) viral hepatitis, (vii) pregnancy or lactation, (viii) prolonged -term excessive drinking. 107 healthy people and 108 T2DM patients were from the First Affiliated Hospital of Jinzhou Medical University. 114 patients with HCC and 112 patients with T2DM combined with HCC were from Tianjin Second People’s Hospital. All T2DM, HCC, and T2DM+HCC patients were newly diagnosed without treatment. Notably, for patients in the T2DM+HCC group, HCC was diagnosed within 1 year after their T2DM diagnosis. We collected the following clinical parameters: gender, age, anthropometric parameters and blood pressure. For blood sample collection, all participants fasted for 12 h before venous blood collection. Blood samples were collected in clot-activator tubes, centrifuged at 2,000 ×g for 10 min to obtain serum, and stored at -80°C for 2 months before analysis.

### Bioinformatics analysis

GM-associated and BA-associated genes were manually curated from the previous reports (15, 16). Diabetes-associated genes were downloaded from DisGeNET (https://www.disgenet.org/search) and the NCBI (17). Differentially expressed genes between normal and HCC tissues were analyzed using RNA-seq data from 3 sources (TCGA-LIHC (18), GSE112790 (19), GSE76427 (20)) *via* R “limma” package (21). To identify intersected targets, we conducted a joint analysis of HCC, T2DM, GM, and BA-associated genes.

### Statistical analysis

Statistical analysis data are expressed as mean ± standard deviation. Single-factor analysis of variance (ANOVA) was used to analyze whether there was a statistical difference between the mean values of different groups. Tukey’s multiple comparison method was used to detect the paired differences between the group mean values to determine which mean values were different. *P* < 0.05 was considered statistically significant.

## Results

### Disease model construction and validation

**Figure 1:**
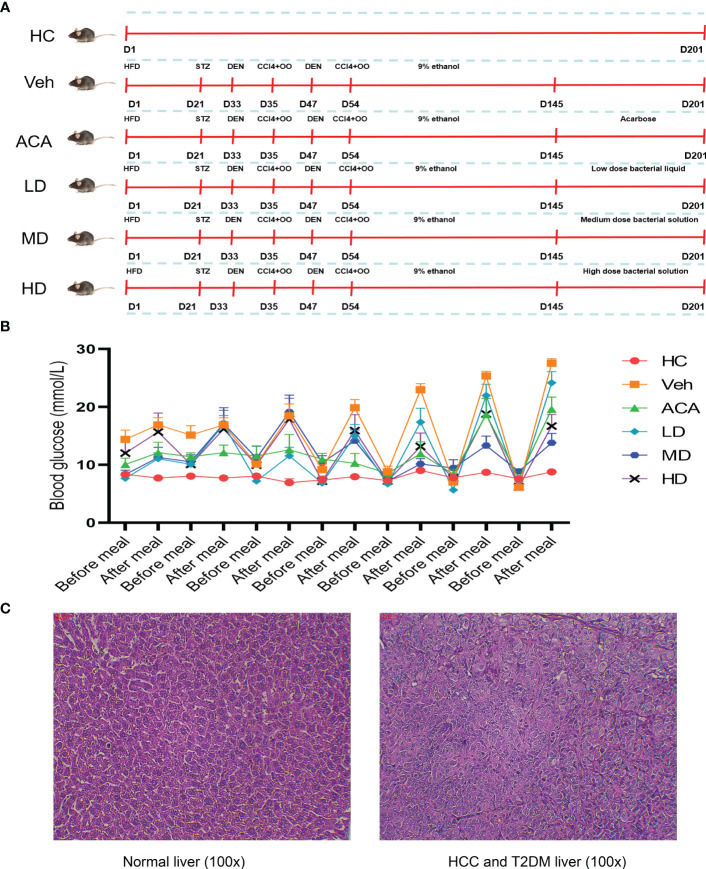
Disease model construction and validation. **(A)** Schematic diagram of animal model construction. 60 mice were randomly assigned to 6 group, health control (HC), vehicle (Veh), acarbose (ACA), low dose *Lactobacillus brevis* (LD), median does *Lactobacillus brevis* (MD), high dose (HD). Indicated drugs were given to establish T2DM and HCC within 180 days. **(B)** Blood glucose measurement before and after meal. Mice were fasting overnight and blood from toes were measured the next morning (before meal) and random measurements were performed within the same day (after meal). **(C)** H&E staining in liver tissue. One mouse was randomly selected for liver tissue sampling from health control and vehicle group. H&E staining was performed to observe the pathological change under microscope (100X).

The mice received a series of treatments to establish T2DM, followed by HCC (**Figure 1A**). Next, we confirmed the model’s success by measuring blood glucose and pathological section. In contrast to health control (HC), all disease mice tested showed high blood glucose values (≥ 11.1 mmol/L) and were considered to have T2DM (**Figure 1B**). Besides, H&E staining indicated nuclear pyknosis, heteromorphic nuclei, and structural disorder, which were all consistent with HCC features (**Figure 1C**).

### *Lactobacillus brevis* stabilizes blood glucose, ameliorates insulin resistance, and alleviates pancreatic and liver damage in mice models

**Figure 2:**
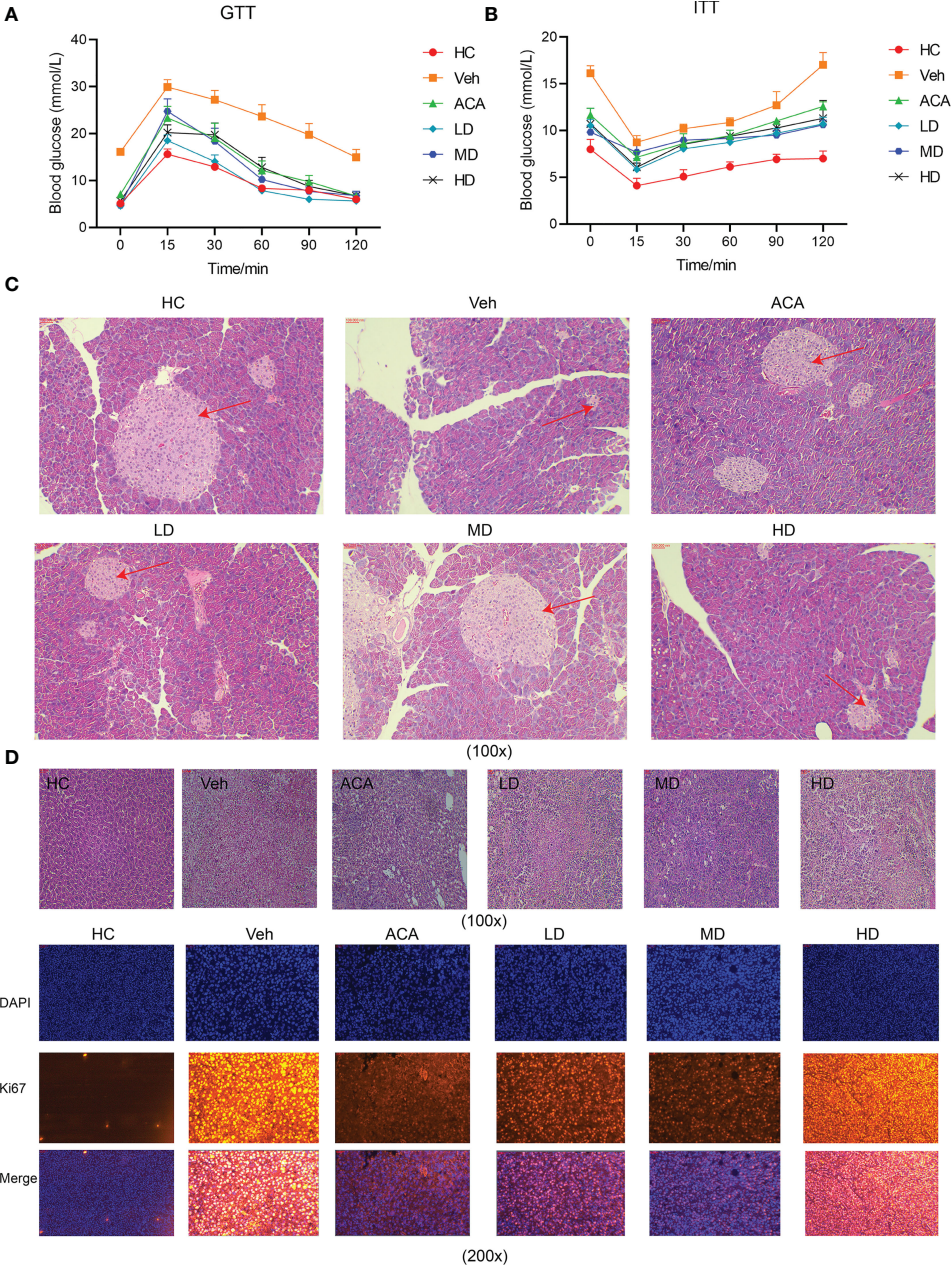
*Lactobacillus brevis* stabilize blood glucose, ameliorate insulin resistance and alleviate pancreatic and liver damage in mice bearing T2DM and HCC. All mice were fasted with indicated time before GTT and ITT experiments. Blood glucose was measured at indicated time points after glucose or insulin injection. **(A)** Blood glucose fluctuation after glucose administration at 15, 30, 60, 90 and 120 minutes. **(B)** Blood glucose fluctuation after insulin administration at 15, 30, 60, 90 and 120 minutes. The mice pancreas and liver tissue were collected for H&E staining and immunofluorescence at the end of experiments. **(C)** The pancreatic area (red arrow) in each group using H&E staining (100X). **(D)** The liver injury (red arrow) in each group using H&E staining (100X) and Ki67 staining in liver tissue using immunofluorescence (100X).

To explore the effect of *Lactobacillus brevis* on disease progression, different dose of live *Lactobacillus brevis* was administered, while ACA was employed as a positive control. We next performed GTT to observe the glucose fluctuation. *Lactobacillus brevis* treatment, regardless of the dose, clearly stabilized the glucose level, suggesting improved pancreatic function (**Figure 2A**). Consistently, insulin resistance showed an apparent amelioration reflected by ITT (**Figure 2B**). Accordingly, H&E staining exhibited alleviated damage to the pancreas (**Figure 2C**). This phenomenon was also observed in liver tissue.

Furthermore, we examined HCC in liver tissue using Ki67, a marker for cell proliferation. Tum cell proliferation was significantly suppressed after *Lactobacillus brevis* treatment (**Figure 2D**). Collectively, *Lactobacillus brevis*, similar to ACA, significantly reduced liver and pancreatic injury, suppressed tumor proliferation, and stabilized blood glucose.

### *Lactobacillus brevis* alleviate GM-associated inflammatory markers and improve prognosis in mice model

**Figure 3:**
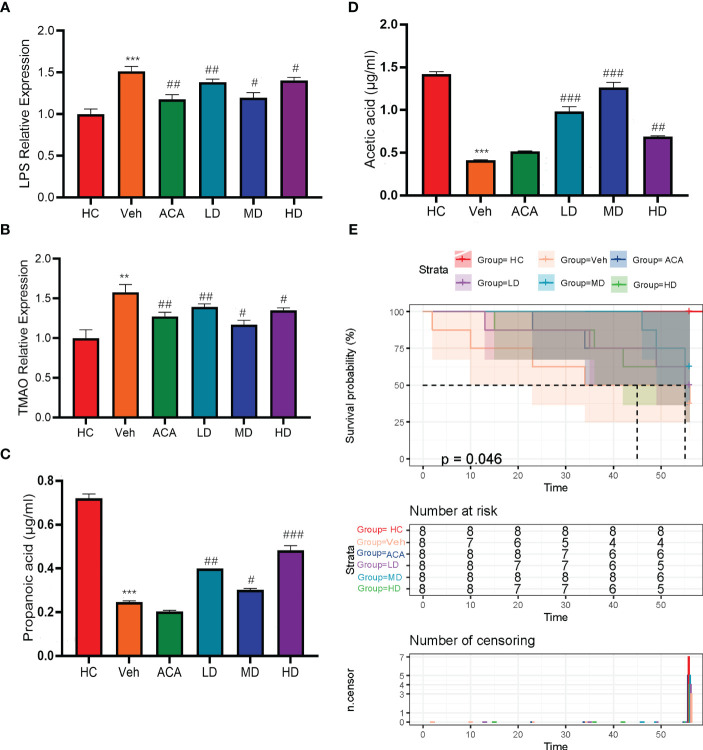
*Lactobacillus brevis* alleviate GM-associated inflammatory markers and improve prognosis in mice bearing T2MD and HCC. **(A, B)** The fold change of LPS and TMAO in each group, related to health control. Comparison between Veh and HC groups (**P < 0.01; ***P < 0.001). Veh was compared with ACA, LD, MD, and HD groups (#P < 0.05; ##: P < 0.01; ###: P < 0.001). **(C, D)** Comparison of differential expression between Propanoic acid and Acetic acid in different groups. Comparison between Veh and HC groups ( ***P < 0.001). # Veh was compared with ACA, LD, MD, and HD groups (#P < 0.05; ##: P < 0.01; ###: P < 0.001). **(E)** Kaplan-Meire plot of mice survival in each group after treatment.

Previous studies indicated the role of *Lactobacillus brevis* in a rodent model for disease improvement by affecting GM diversity (22). Furthermore, it is widely accepted that GM can positively or negatively impact host metabolism *via* GM-associated inflammatory markers, including short-chain fatty acids (SCFAs), lipopolysaccharides (LPS), and trimethylamine N-oxide (TMAO) (23). In our experiment, we thus examined LPS and TMAO levels, as they were parameters associated with disease severity. Consistently, mice with T2DM+HCC displayed higher levels of both markers than the normal group. However, both ACA and probiotic treatment strikingly reduced the inflammation level, confirming the role of GM in affecting disease progression *via* reshaping the metabolic spectrum (**Figures 3A, B**; **Supplement Table 2**). We also examined SCFAs and compared them with the Veh group. Propanoic acid and Acetic acid expression levels were significantly increased after intervention with probiotics and acarbose (**Figures 3C, D**). Moreover, the prognosis, reflected by the death number, was improved in probiotic groups (**Figure 3E**). Collectively, *Lactobacillus brevis* dampened the GM-associated inflammation and improved prognosis.

### *Lactobacillus brevis* affect the diversity of the gut microbial community

**Figure 4:**
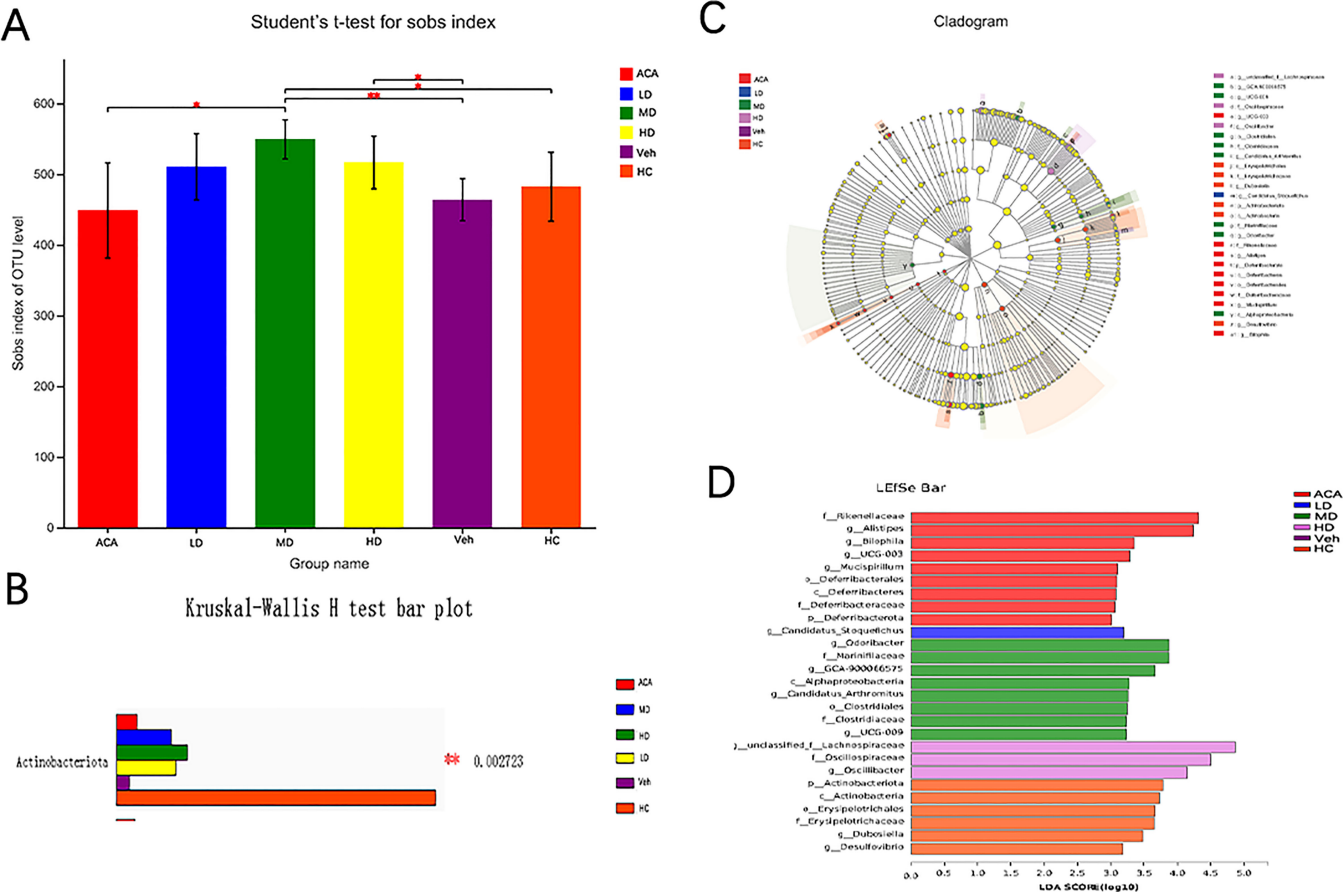
*Lactobacillus brevis* affect diversity of gut microbial community 16SrDNA sequence were performed from 5 mice in each group with fecal samples. **(A)** Alpha diversity in each group. Sobs index was quantified to reflect the GM diversity, student’s t-test; The closer the 2 sample points are, the more similar their species composition is. The horizontal and vertical coordinates represent the relative distance. **(B)** Multi-species comparison histogram. **(C)** LEfSe multilevel species difference discriminant analysis. **(D)** LDA score. LDA is used to estimate each species' abundance impact on the different effects. * P<0.05; ** P<0.01.

As indicated by the changes in GM-associated markers, we next performed a 16SrDNA sequence to understand the GM components after treatment. Alpha diversity analysis determined the community diversity of specific habitats, and the Sobs index reflects the richness of the community (**Figure S1A**). We found that *Lactobacillus brevis* significantly changed the GM diversity, regardless of the probiotic dose (**Figure 4A**). Meanwhile, beta diversity analysis confirmed a distinct GM clustering between normal and disease groups, visualized by PCA, PcoA, and NMDS (**Figures S1B–D**). Both analyses indicated the different species community composition in normal and disease groups and distinctions between disease groups. We further interrogated the distribution and typing analysis of the dominant bacteria. At the phyla level, the major GM components were reshaped to *Actinomycetes, Alistipes, Bacteroides*, *Desulfovibrio*, *Dubosiella*, and *Firmicus* after *Lactobacillus brevis* treatment (**Figure 4B**). This was consistent with the composition of GM at the genus level (**Figure S1E**). To further narrow down the findings, multilevel species difference discriminant analysis was used to estimate the different impacts of species abundance on each group (**Figure 4C**). *Actinomycetes* were significantly reduced in the disease group compared with the healthy control. However, the quantity of *Actinomycetes* has partially restored after ACA and *Lactobacillus brevis* intervention (**Figure 4D**). Altogether, *Lactobacillus brevis* significantly changed the GM diversity, and *Actinomycetes* was identified as a potential interest in disease progression.

### TBA level is increased in patients with T2DM+HCC but reduced in mice after Lactobacillus brevis intervention

**Figure 5:**
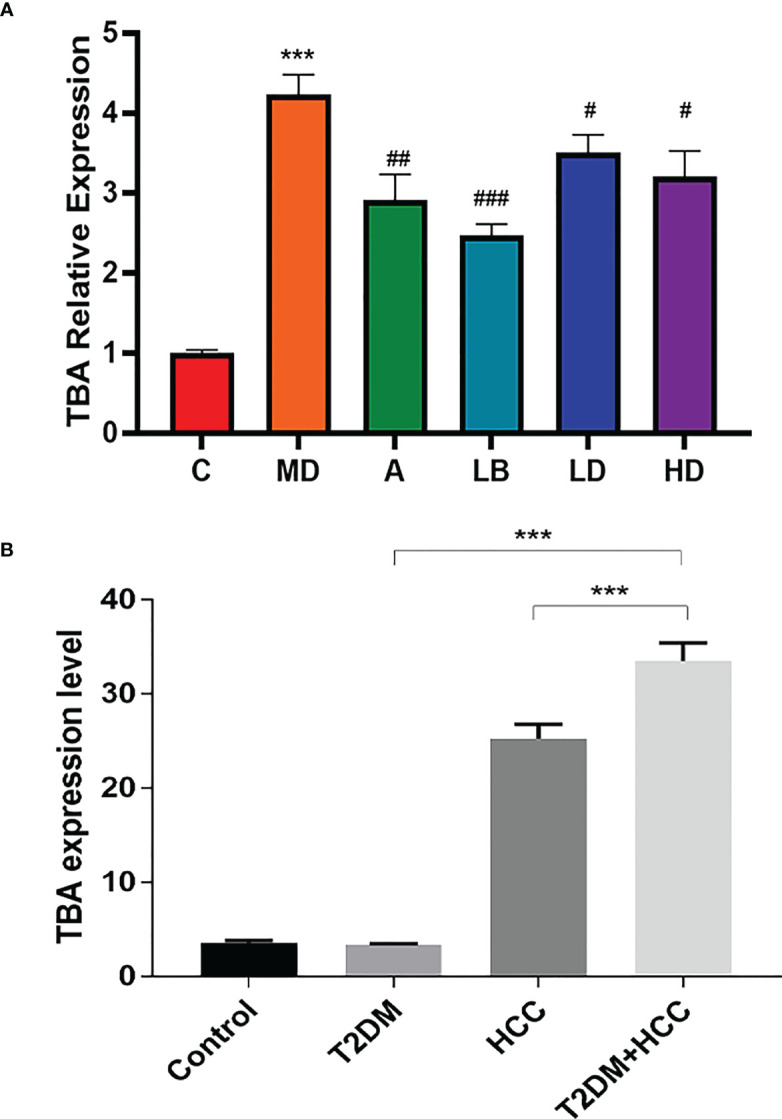
TBA level is increased in patients with T2DM+HCC but reduced in mice after Lactobacillus brevis intervention. TBA was measured using ELISA from blood samples from clinical patients and mice model. **(A)** The fold change of TBA in each mice group, the value was related to health control. Comparison between Veh and HC groups (***P < 0.001). Veh was compared with ACA, LD, MD, and HD groups (#P < 0.05; ##P < 0.01; ###P < 0.001). **(B)** The TBA levels in clinical cohorts (health, T2DM, HCC and T2DM+HCC). Comparison between Veh and HC groups (***P < 0.001).

TBA is a critical clinical parameter in evaluating T2DM+HCC severity (24). Previous studies verified that GM, including *Actinomycetes, Bacteroides*, and *Firmicus*, could regulate tumor progression through BA metabolism (25). Our analysis also observed the elevated TBA in three disease groups compared with normal control. However, *Lactobacillus brevis* intervention reduced TBA level, associated with better prognosis (**Figure 5A**). Therefore, we further investigated the TBA in the clinical scenario. We included 107 participants in the healthy group (85 males and 22 females), 108 participants with T2DM (95 males and 13 females), 114 participants with HCC (86 males and 26 females), and 112 participants with T2DM + HCC (75 males and 37 females). There were no significant differences in gender, fasting plasma glucose (FPG), creatinine (Cr), and uric acid (Ua) among the 4 groups. As expected, subjects with HCC or T2DM + HCC had significantly higher gamma-glutamyl transferase (r-GT), alkaline phosphatase (ALP), alpha-fetoprotein (AFP), total bilirubin (TBIL), and direct bilirubin (DBIL) than those in the healthy population group, indicating the liver function damage. In contrast, albumin (ALB), total cholesterol (TC), high-density lipoprotein (HDL), and low-density lipoprotein (LDL) levels were significantly lower. Moreover, patients with HCC or T2DM + HCC had significantly higher TBA levels compared to the T2DM group (**Table 1**). We also performed a correlation analysis to seek the potential link between GM and metabolic parameters. We found several key GM communities interfered with by *Lactobacillus brevis* were clearly associated with rodent blood glucose, TBA level, and human TBAs (**Figure 5B**). TBA level was increased in clinical and preclinical samples, whereas *Lactobacillus brevis* alleviated TBA growth potentially through interfering GM in the mice model.

**Table 1:** Anthropometric parameters and biochemical indexes among subjects with control, T2DM, HCC, and T2DM+HCC.

Variables	Control (n = 107)	T2DM (n =108)	HCC (n = 114)		T2DM+HCC (n = 112)	P
Male/Female	85/22	95/13	86/26		75/37	0.458
Age (Years)	49.83 ± 8.10	50.61 ± 13.61	59.27 ± 0.802		58.31 ± 10.36	0.003
BMI (Kg/m2)	23.98 ± 2.46	26.31 ± 3.21	23.13 ± 3.46		26.01 ± 2.68	0.010
FPG (Kg/m2)	5.19 (4.87-5.51)	9.01 (7.31-10.92)	4.41 (4.08-5.08)		7.36 (6.21-11.76)	0.116
ALT (u/L)	27 (16-35)	37 (20-55)	53 (32-95)^*^		39 (29-88)	0.001
r-GT (u/L)	27.2 (19.8-35.6)	29 (20-48)	116 (58-223)^*^		145 (98-252)^*#^	<0.001
ALP (u/L)	67 (57-85)	82 (65-109)	123.5 (88.98-158)^*#^		145 (64-251)^*#^	<0.001
ALB (g/L)	46.7 (44.1-47.2)	42.8 (40.1-45.2)^*^	40.44 (38.75-42.13)^*#^		35 (31.26-39.15)^*#^	<0.001
TBA (umol/l)	3.80 (2.60-4.90)	3.20 (2.30-4.50)	23.94 (16.6-31.28)^*#^		30 (22.60-34.90)^*#^	<0.001
TBIL (umol/l)	13.9 (11.1-19.7)	16.1 (10.60-19.90)	32.99 (23-42.99)^*#^		23 (12.60-34.90)^*#^	<0.001
DBIL (umol/l)	3.7 (3.1-5.2)	4.5 (2.60-5.90)	17.12 (8.792-25.46)^*#^		19.2 (12.60-24.90)^*#^	<0.001
Cr (umol/l)	61.23 ± 13.21	57.56 ± 12.36	68.6 ± 2.183		61.35 ± 12.54	0.965
UA (umol/l)	290 (220-321)	264 (222.60-3154.90)	306.6 (289.7-323.6)		233 (212.30-314.40)	0.612
eGFR (ml/min/1.73m2)	89.7 (81.23-107.2)	103.2 (82.60-94.90)	97.45 (94.45-100.5)^*^		113.06 (42.60-74.90)^*^	<0.001
TC (mmol/l)	4.26 ± 0.71	5.62 ± 0.91	0.961 ± 0.04^*#^		3.65 ± 0.56^*#^	0.005
TG (mmol/l)	0.91 (0.71-1.31)	1.37 (0.76.-2.90)	1.25 (0.63-1.32)^*^		1.32 (0.60-1.56)^*^	0.013
HDL (mmol/l)	1.41 ± 0.41	1.10 ± 0.32	1.22 ± 0.03		1.30 ± 0.12	<0.001
LDL (mmol/l)	2.81 ± 0.52	3.02 ± 0.42	2.03 ± 0.07^*#^		2.01 ± 0.75^*#^	<0.001
AFP (ng/ml)	2.63 (2.03-4.21)	3.62 (2.30-4.50)	362.6 (220.9-504.2)^*#^		395 (322.60-554.90)^*#^	<0.001
CEA (ng/ml)	3.18 (1.88-5.62)	6.13 (1.60-9.65)^*^	6.172 (2.354-9.99)^*^		6.51 (2.30-9.90)^*^	<0.001

* for comparison to the control group, # for comparison to T2DM or HCC.

### MMP9 is bioinformatically identified as a target associated with BA metabolism in T2DM+HCC

**Figure 6:**
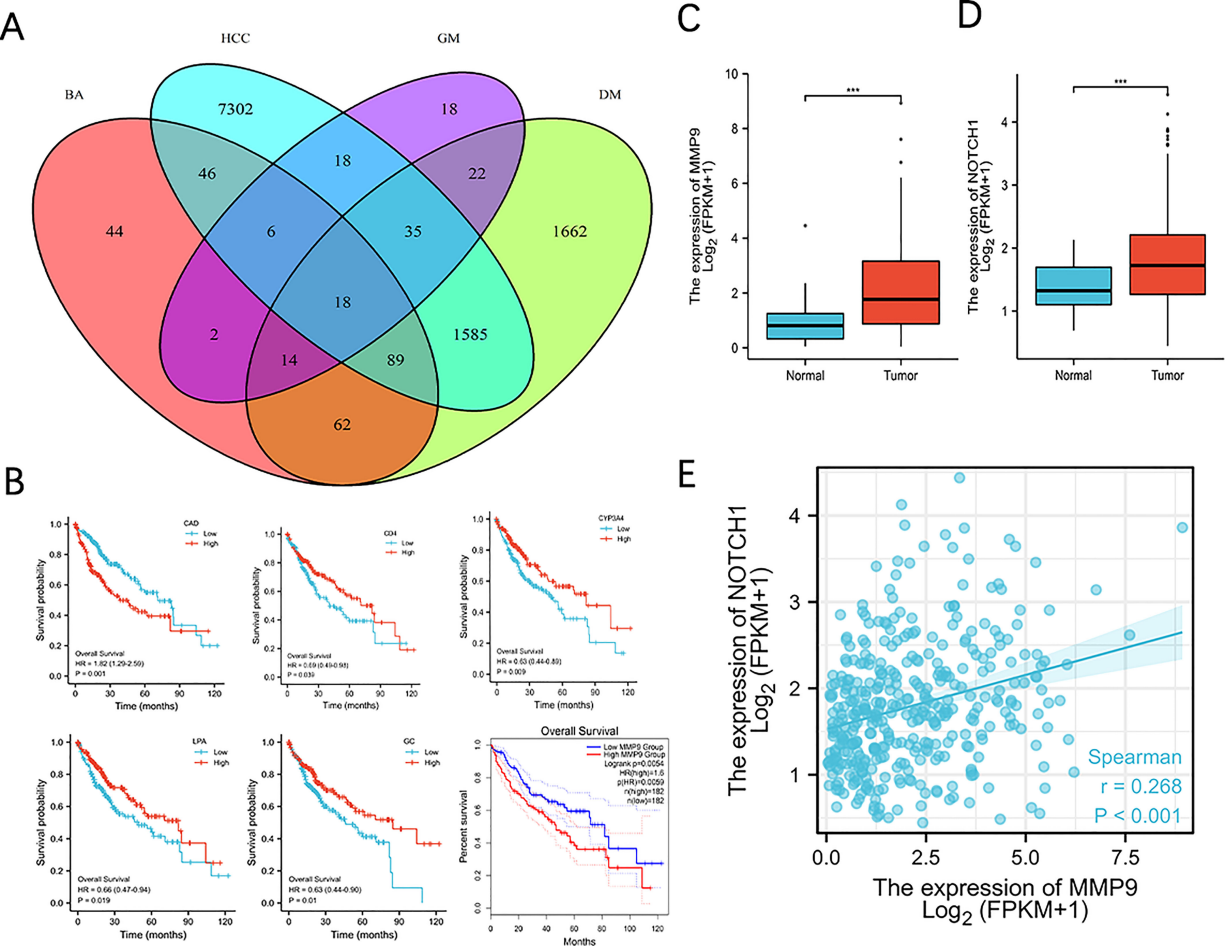
MMP9 is bioinformatically identified as a target associated with BA metabolism in T2MD+HCC. **(A)** Venn diagram on identifying 18 core candidates. Differentially expressed genes in HCC (vs. health) from three datasets were intersected with DM-, GM- and BA-associated genes. **(B)** Survival curve of 6 genes in HCC. Kaplan Meire analysis was performed using the 18 genes identified and genes exhibited statistic significance was demonstrated. **(C, D)** MMP9 and Notch 1 expression in normal and HCC group. *** P < 0.001. **(E)** Correlation analysis of MMP9 and Notch1 expression.

To identify the potential molecular mechanism, we adopted a combined approach by intersecting differentially expressed genes (HCC vs. Normal), DM-associated genes, GM-associated genes and BA-associated genes. We identified 18 genes (**Figure 6A**). To narrow the range, we further performed a Kaplan-Meier plot and found 6 genes associated with HCC prognosis (**Figure 6B**). We selected MMP9 for further validation as it was reported as an oncogene in multiple cancer types, including HCC, and BA treatment could promote MMP9 expression (26). Moreover, MMP9 expression was significantly upregulated in HCC compared with the normal group (**Figure 6C**). Several studies indicated the overexpression of MMP9 in promoting NOTCH 1 signaling to affect tumorigenesis (27, 28). Consistently, we found that NOTCH 1 expression was higher in the HCC group (**Figure 6D**) and was positively correlated with MMP9 (**Figure 6E**).

### *Lactobacillus brevis* affect MMP9 expression and NOTCH signaling in mice bearing T2DM + HCC

**Figure 7:**
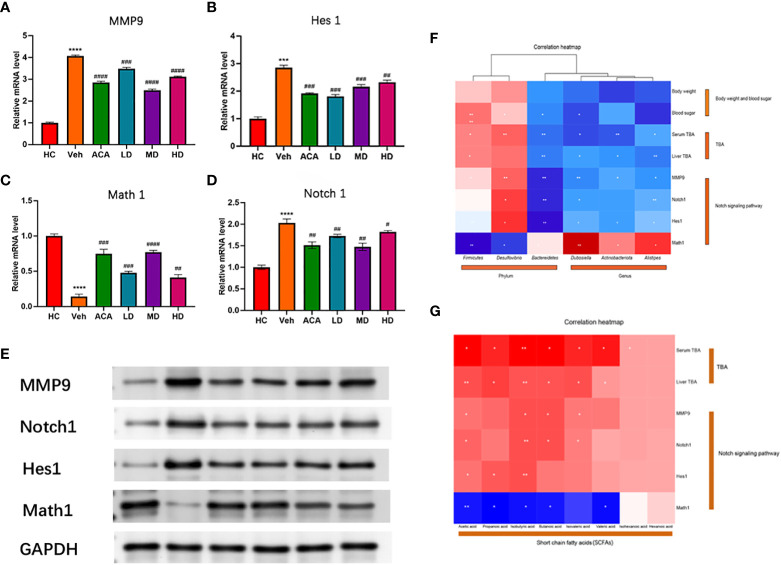
*Lactobacillus brevis* affect MMP9 expression and Notch1 signaling in mice bearing T2DM + HCC. **(A–D)** mRNA level of MMP9, Notch1, Hes1 and Math1. The transcripts were quantified by qPCR from mice liver tissue. Compare the HC group with the Veh group (***: P<0.001; ****: P<0.0001). Comparison of Veh group with ACA, LD, MD, and HD groups (#: P<0.05; ##: P<0.01; ###: P<0.001; ####: P<0.0001). **(E)** The protein level of MMP9, Notch1, Hes1 and Math1. Western blot was performed to measure the protein level using mice liver tissue. **(F, G)** Correlation analysis of MMP9, Notch1, Hes1 and Math1 with GM and SCFA. *: P<0.05; **: P<0.01.

To verify the bioinformatic findings, we performed qPCR to examine the expression of MMP9, NOTCH 1 and its two downstream transcription factors, Hes1 and Math1. MMP9, NOTCH 1, and Hes1 decreased in the probiotic and ACA intervention groups. The disease group overall displayed higher expression of these four transcripts. However, *Lactobacillus brevis* reduced their expression, consistent with Western blot findings (**Figures 7A–E**). Interestingly, these molecular markers were strongly associated with GM richness interfered with by *Lactobacillus brevis* (**Figure 7F**) and SCFAs (**Figure 7G**). Lactobacillus brevis may prevent disease progression by inhibiting MMP9 and NOTCH 1 signaling.

## Discussion

T2DM+HCC are highly prevalent and intricate diseases worldwide. Epidemiological studies have demonstrated a significant increase in the risk of developing certain tumors in T2DM patients. The comorbidity risk is attributed to multifactorial changes in genes and epigenetics, such as epigenetic modifications, transcription and translation alterations, and signaling pathway modifications (29). From a clinical standpoint, a substantial increase in TBA was observed in untreated patients with T2DM+HCC. In our study, through 16S rDNA sequence and bioinformatics analysis, we identified *Lactobacillus brevis* could reshape the GM diversity and metabolic landscape (like BA and SCFAs). We further predicted and validated that *MMP9* and *Notch* signaling are crucial mechanisms to mediate disease progression, which could be dampened by probiotic intervention. Our study suggested a complex interplay network among GM, metabolites, and oncogenic pathways.

Prior studies have demonstrated that *Lactobacillus brevis* can lessen blood glucose and body weight in T2DM mice induced by STZ and a high-fat diet. Moreover, reduced breast cancer progression was also observed (30). It is reasonable to infer that probiotic intervention may remotely function through regulating GM and relevant metabolites to prevent carcinogenesis and tumorigenesis. Considering that the liver is the major organ receiving GM-associated metabolites through the gut-liver axis, it would be interesting to explore whether probiotic treatment could be a potential option to limit HCC progression in patients with T2DM. However, to the best of our knowledge, there is no study focusing on probiotic treatment for T2DM+HCC.

This study established the T2DM model using a high-fat diet and STZ induction. We then utilized DEN and CCL_4_ to develop HCC in the T2DM model. ACA was given as a positive control. We confirmed that *Lactobacillus brevis*, regardless of concentration, can effectively improve blood glucose levels and insulin resistance in mice beating T2DM+HCC. The liver and pancreatic pathological and inflammatory/metabolic indicators, such as TBA, LPS, and TMAO, further supported this. These results align with the anti-cancer effects of *Lactobacillus brevis* observed on the gastric cancer cell line (31).

In recent years, many studies have shown that GM participates in host metabolism and is closely related to the occurrence and development of T2DM (32). It is not surprising that GM intervention may affect T2DM progression and therapeutic effects both positively and negatively (33). Our study confirmed the previous findings where *Lactobacillus brevis* stabilize blood glucose and alleviate insulin resistance, together with less injury to the pancreas. Metabolites, generated or processed by GM, enter the liver through the gut-liver axis. It, therefore, may reversely affect liver function. Thus, we performed a 16SrDNA sequence and identified several significant GM changes in the disease model. *Bacteroides* and *Firmicutes* dominate the GM of healthy adults (34). Zhang et al. showed that compared with the average population, the number of *Bacteroides* in T2DM+HCC patients decreased significantly (35). Xie et al. found that the content of *Bacteroides* and *Lactobacillus* in T2DM mice was significantly reduced (36).

Moreover, *Actinomycetes* richness is decreased in the disease model but partially restored after the probiotic intervention, showing the same trend with disease improvement and prognosis. Interestingly, *Actinomycetes* richness was also increased after surgery in patients with gastric cancer (33), and *Actinomycetes* extracts showed bioactive effects on cancer-related signaling pathways, such as Wnt and hedgehog (37). Accordingly, verifying whether Lactobacillus brevis-induced therapeutic effect is mediated by Actinomycetes and its metabolites would be reasonable.

BAs are synthesized in the liver and secreted into the intestine. The composition of GM can alter BAs metabolism, leading to changes in the production of secondary BAs from primary BAs (38). Dysregulated components may directly contribute to BA metabolic disorder (39). It is rational to infer that GM diversity may regulate T2MD and HCC progression and therapeutic response through balancing or disrupting BA metabolism. A recent study has shown that fecal microbiota and BAs are associated with immunotherapy outcomes of unresectable HCC (40). In this study, we observed higher levels of TBA in both clinical patients and pre-clinical mice models. Our results confirmed the previous findings. Moreover, *Lactobacillus brevis* intervention decreased the TBA levels, and TBA is correlated with the GM components, suggesting the interaction between GM and BA metabolism.

To seek potential molecular mechanisms mediating the disease improvement, we bioinformatically intersected gene candidates generated from differential analysis, Diabetes- BA- and GM-associated genes, and we identified MMP9 and NOTCH 1 signals, of which both are widely reported to promote HCC progression (41, 42). Lin et al. showed that Delta-like 1 homolog (*DLK1*), a transmembrane and secreted protein, can regulate NOTCH 1 signaling by upregulating MMP9 expression, thereby promoting the invasion of lung cancer (43). In addition, overexpression of *SLC6A8* promotes proliferation, migration, and invasion of non-small cell lung cancer, accompanied by upregulation of *MMP9* and activation of the NOTCH 1 signaling pathway (27). Our model also observed upregulation of MMP9 and NOTCH1 at transcriptional and translational levels. Recent studies have found that *MATH1* is a tumor suppressor gene, and its abnormal expression is involved in the occurrence and development of various cancers (44, 45). Leow et al. confirmed that *Math1* could inhibit the proliferation of colon cancer cells by up-regulating *P27* and down-regulating *cyclinD1* expression (46). Consistently, we also observed that Math1 is downregulated by *Lactobacillus brevis* treatment. Correlation analysis further establishes the link between TBA, GM components, and molecular targets. This indicates a complex interplay among GM, metabolites, and molecular oncogenes.

## Limitations

In this study, we conducted a correlation analysis among the GM, inflammation/metabolites, and NOTCH 1 signaling pathways to speculate that probiotics may affect the disease. However, we have not confirmed the direct impact of the changed GM components or BAs on HCC and T2DM progression. Therefore, our future efforts will investigate the mechanism of T2DM+HCC progression based on a specific bacterium or metabolite.

## Conclusions

This study explores the therapeutic effect of *Lactobacillus brevis* in improving T2DM+HCC potentially through the GM-BA-liver axis *via* regulating oncogenic signaling pathways in mice models. We systematically analyzed the differences in GM, SCFAs, and TBAs in the T2DM+HCC model after drug and probiotic interventions. We found that MMP9 and NOTCH signaling may serve as the mechanism to connect GM and BA crosstalk. Our study could support the new therapeutic option for T2DM+HCC based on GM intervention.

## Data availability statement

The datasets presented in this study can be found in online repositories. The names of the repository/repositories and accession number(s) can be found in the article/**Supplementary Material**.

## Ethics statement

The human experiments have been approved by the Medical Ethics Committee of Tianjin Second People’s Hospital, with approval number [2021]52 of the Tianjin Medical Ethics Review. The patients/participants provided their written informed consent to participate in this study. According to the animal ethics statement, approved by Beijing Weishanglide Biotechnology Co., Ltd. (No.VS212600898), animal experiments have been conducted.

## Author contributions

SC, QZ, and PH collaborated on the experiment design. JLiu, PL, and PH assisted with data acquisition and analysis. SC and PH were responsible for writing the manuscript, which was reviewed by JLi, LG, and LZ, who contributed equally to the work. All authors contributed to the article and approved the submitted version.

## Supplementary material

Supplementary Figure 1
*Lactobacillus brevis* affect diversity of gut microbial community 16SrDNA sequence were performed from 5 mice in each group with fecal samples. **(A)** Sobs index in each group, reflecting the richness of the GM community; **(B–D)** PCA, PCoA and NMDS on OTU level. **(E)** The clustering indicated the similarities within and between each group. The clustering indicated the similarities within and between each group.
