# Supplementary figures and images for: Lactobacillus brevis alleviates the progress of hepatocellular carcinoma and type 2 diabetes in mice model via interplay of gut microflora, bile acid and NOTCH 1 signaling
Source: Front Immunol. 2023 May 10;14:1179014. doi: 10.3389/fimmu.2023.1179014 (PMC10206262; doi:10.3389/fimmu.2023.1179014)

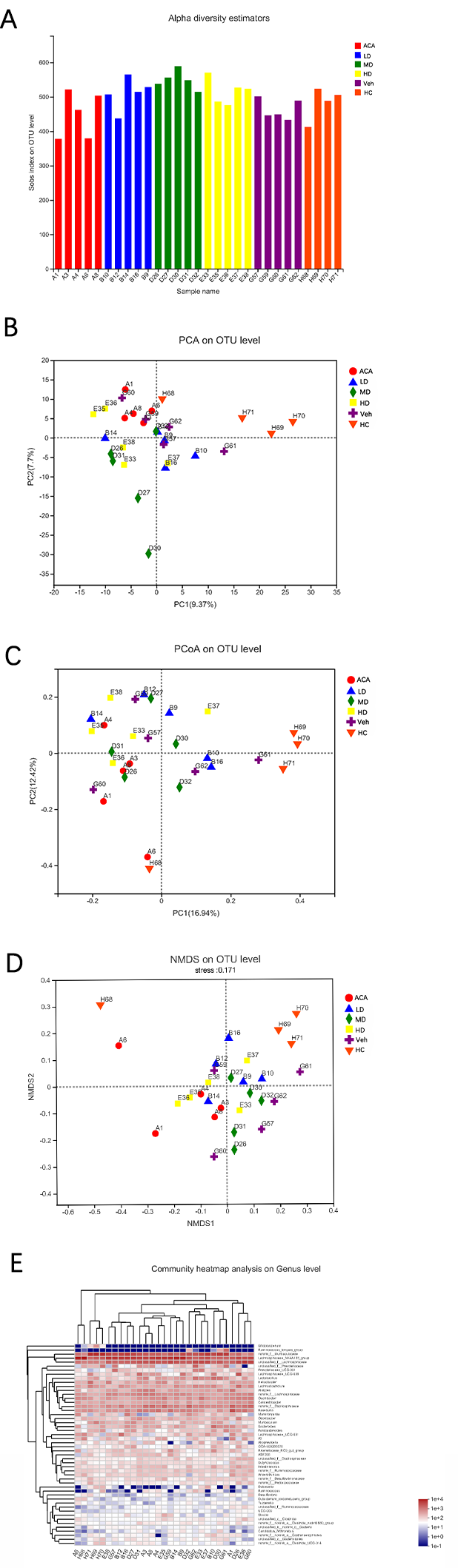

Supplement: Supplementary Figure 1 — Lactobacillus brevis affect diversity of gut microbial community 16SrDNA sequence were performed from 5 mice in each group with fecal samples. (A) Sobs index in each group, reflecting the richness of the GM community; (B–D) PCA, PCoA and NMDS on OTU level. (E) The clustering indicated the similarities within and between each group. The clustering indicated the similarities within and between each group. [file Image_1.tif]
