# Supplementary material for: Lactobacillus brevis alleviates the progress of hepatocellular carcinoma and type 2 diabetes in mice model via interplay of gut microflora, bile acid and NOTCH 1 signaling
Source: Front Immunol. 2023 May 10;14:1179014. doi: 10.3389/fimmu.2023.1179014 (PMC10206262; doi:10.3389/fimmu.2023.1179014)
Supplement: Supplementary file 2 [file Table_1.docx]

**Table 1 Primer sequence**

| Genes | Primer sequences |
| --- | --- |
| Notch1 | F: GATGGCCTCAATGGGTACAAG  R: TCGTTGTTGTTGATGTCACAGT |
| Hes1 | F: TCAACACGACACCGGACAAAC  R: ATGCCGGGAGCTATCTTTCTT |
| Math1 | F: GAGTGGGCTGAGGTAAAAGAGT  R: GGTCGGTGCTATCCAGGAG |
| MMP9 | F: TCTATGGTCCTCGCCCTGAA  R: TTGTATCCGGCAAACTGGCT |
