# Supplementary material for: Lactobacillus brevis alleviates the progress of hepatocellular carcinoma and type 2 diabetes in mice model via interplay of gut microflora, bile acid and NOTCH 1 signaling
Source: Front Immunol. 2023 May 10;14:1179014. doi: 10.3389/fimmu.2023.1179014 (PMC10206262; doi:10.3389/fimmu.2023.1179014)
Supplement: Supplementary file 3 [file Table_2.docx]

Table 2: Expression differences of TBA, LPS, and TMAO under different intervention measures.

| Groups | TBA (μmol/L) | LPS (EU/L) | TMAO (mmol/L) |
| --- | --- | --- | --- |
| HC | 6.19±0.21 | 7.28±0.36 | 61.95±5.39 |
| Veh | 26.22±1.23^a^ | 11.01±0.34^a^ | 97.77±4.96^a^ |
| ACA | 18.07±1.58^b^ | 8.56±0.33^b^ | 78.97±2.62^b^ |
| MD | 15.32±0.71^b^ | 8.71±0.36^b^ | 72.47±2.75^b^ |
| LD | 21.69±1.15^b^ | 10.07±0.21^b^ | 86.24±1.95^b^ |
| HD | 19.84±1.62^b^ | 10.21±0.22^b^ | 83.67±1.53^b^ |

Note: "a" indicates statistical significance compared to the HC group (*P* < 0.05), "b" indicates statistical significance compared to the Veh group (*P* < 0.05). LPS, lipopolysaccharide; TMAO, trimethylamine N-oxide. TBA, total bile acid.
